# Cerebrovascular G_i_ Proteins Protect Against Brain Hypoperfusion and Collateral Failure in Cerebral Ischemia

**DOI:** 10.1007/s11307-022-01764-8

**Published:** 2022-09-08

**Authors:** Salvador Castaneda-Vega, Sandra Beer-Hammer, Veronika Leiss, Hanna Napieczyńska, Marta Vuozzo, Andreas M. Schmid, Hang Zeng, Yi He, Ursula Kohlhofer, Irene Gonzalez-Menendez, Leticia Quintanilla-Martinez, Johann-Martin Hempel, Maik Gollasch, Xin Yu, Bernd J. Pichler, Bernd Nürnberg

**Affiliations:** 1 Werner Siemens Imaging Center, Department of Preclinical Imaging and Radiopharmacy, Eberhard Karls University Tübingen and University Clinic, Tübingen, Germany; 2 Department of Nuclear Medicine and Clinical Molecular Imaging, Eberhard Karls University Tübingen and University Clinic, Tübingen, Germany; 3 Department of Pharmacology, Experimental Therapy and Toxicology, and Interfaculty Center for Pharmacogenomics and Drug Research, Eberhard Karls University Tübingen and University Clinic, Tübingen, Germany; 4 Cluster of Excellence iFIT (EXC 2180) “Image-Guided and Functionally Instructed Tumor Therapies”, Eberhard Karls University Tübingen and University Clinic, Tübingen, Germany; 5 High Field Magnetic Resonance Department, Max Planck Institute for Biological Cybernetics, Tübingen, Germany; 6 Institute of Pathology and Neuropathology, Eberhard Karls University and Comprehensive Cancer Center, Eberhard Karls University Tübingen and University Clinic, Tübingen, Germany; 7 Department of Diagnostic and Interventional Neuroradiology, Eberhard Karls University Tübingen and University Clinic, Tübingen, Germany; 8 Experimental and Clinical Research Center (ECRC), A Joint Cooperation Between the Charité Medical Faculty and the Max Delbrück Center for Molecular Medicine in the Helmholtz Association (MDC), Berlin, Germany; 9 Department of Internal and Geriatric Medicine, University – Medicine Greifswald, Greifswald, Germany; 10 Athinoula A. Martinos Center for Biomedical Imaging, Massachusetts General Hospital and Harvard Medical School, Charlestown, MA USA

**Keywords:** Ischemic stroke, MRI, Pertussis toxin, G protein, Cerebral microvessels

## Abstract

Cerebral hypoperfusion and vascular dysfunction are closely related to common risk factors for ischemic stroke such as hypertension, dyslipidemia, diabetes, and smoking. The role of inhibitory G protein-dependent receptor (G_i_PCR) signaling in regulating cerebrovascular functions remains largely elusive. We examined the importance of G_i_PCR signaling in cerebral blood flow (CBF) and its stability after sudden interruption using various *in vivo* high-resolution magnetic resonance imaging techniques. To this end, we induced a functional knockout of G_i_PCR signaling in the brain vasculature by injection of pertussis toxin (PTX). Our results show that PTX induced global brain hypoperfusion and microvascular collapse. When PTX-pretreated animals underwent transient unilateral occlusion of one common carotid artery, CBF was disrupted in the ipsilateral hemisphere resulting in the collapse of the cortically penetrating microvessels. In addition, pronounced stroke features in the affected brain regions appeared in both MRI and histological examination. Our findings suggest an impact of cerebrovascular G_i_PCR signaling in the maintenance of CBF, which may be useful for novel pharmacotherapeutic approaches to prevent and treat cerebrovascular dysfunction and stroke.

**Supplementary Information:**

The online version contains supplementary material available at 10.1007/s11307-022-01764-8.

## Introduction

G_i_ proteins are the principal signal transducers of a broad subset of G protein-coupled receptors (termed G_i_PCRs), including those for acetylcholine, adenosine, and catecholamines that control blood circulation [1–5]. In spite of many studies, the functions of G_i_ protein-dependent signaling in the brain vasculature have been largely ignored. One major reason for the undefined role of G_i_ proteins may be the lack of appropriate animal models, since a simultaneous genetic ablation of the major Gα_i_ isoforms (Gα_i2_ and Gα_i3_) produces embryonic lethality in mice [6]. On the other hand, the significance of singular knockouts is limited by overlapping functions of these isoforms. Pertussis toxin (PTX) has been used to study G_i_ protein signaling in the cardiovascular system [7, 8], but the effects on cerebrovasculature have not yet been elucidated.

*In vivo*, PTX irreversibly and with high specificity blocks G_i_-linked GPCR signal transduction — hereafter referred to as non-cerebral G_i_PCR KO — by catalyzing covalent modification of a C-terminal cysteine residue of cellular Gα_i_ isoforms [9–11]. In arteries and microvessels, PTX inhibits endothelium-dependent relaxation to certain agonists such as β-adrenergic ligands, angiotensin, serotonin, or relaxins and is therefore useful for the study of vasculopathies [2, 7, 8, 12–18]. We have previously demonstrated that PTX, administered in a single peritoneal injection, does not cross the blood–brain barrier (BBB) and does not modify G_i_PCR signaling in neurons [19]. PTX administration, however, still irreversibly interrupts G_i_PCR signaling for up to 96 h in cells outside of the CNS, including brain vasculature. Using PTX, in this work, we evaluate the effects in cerebral blood flow caused by permanent G_i_PCR modification of cells outside of the brain.

MRI provides a powerful set of neuroimaging tools that allow quantification of pathological changes at the functional level in the brain [20–22]. *In vivo* MRI allows consistent acquisition of multiple tissue characteristics in a single session permitting evaluation of their longitudinal development [20, 22].

In the present study, we focused, first, on the effects of PTX administration in systemic blood flow, cerebral blood flow (CBF), and microvascular patency and, second, on G_i_PCR-dependent vascular responses after acute vascular occlusion. Our data reveal that injection of PTX severely reduces cerebral perfusion and impedes compensatory mechanisms regulating CBF. As a result, microcirculation collapses during vascular occlusion, contributing to ischemic brain lesions.

## Materials and Methods

### Animal Experiments

The study was carried out in compliance with the ARRIVE guidelines. All experiments were performed according to the EU Animals Scientific Procedures Act and the German law for the welfare of animals and were approved by the local animal ethical committees (Regierungspräsidium Tübingen, PH 10/13, PH 1/11, PH 04/19). C57BL/6 female, 8-week-old, mice were kept under specified pathogen-free conditions, controlled temperature, and humidity in 12-h day/night light cycles, receiving food and water ad libitum. Workflows of all experiments are sketched in the corresponding figures. We induced non-cerebral G_i_PCR KO using a single dose of PTX (150 μg/kg body weight) (Merck, Darmstadt, Germany) 48 h before intervention (sham or surgery), as previously shown [19, 23, 24]. Details are provided in the “Supplementary Information.”

### Whole-Body Semi-quantitative Perfusion

In order to determine whether PTX produced whole-body systemic organ-perfusion changes, mice weighing 20–23 g were evaluated using dynamic contrast-enhanced (DCE) MRI. Vehicle (phosphate-buffered solution, PBS) or PTX was injected intraperitoneal (i.p.) into 5 mice per group. After 48 h, transversal DCE images focusing on multiple body regions including the lung, kidney, paravertebral muscle, abdominal vessels, heart, and brain were acquired. Details are provided in the “Supplementary Information.”

### Longitudinal Multiparametric MRI—Animals and Treatment

For these experiments, 8-week-old mice were divided into four groups: PBS-pretreated sham-operated, PTX-pretreated sham-operated, PBS-pretreated occluded, and PTX-pretreated occluded. These groups were studied in two main experimental settings: in the first setting, mice were imaged immediately after vessel occlusion using a non-absorbable suture around one common carotid artery (CCA) or a sham surgery. In the second setting, the groups were imaged 1 week before (baseline) and 48 h after a transient occlusion (lasting 30 min) of one CCA or a sham surgery. Further experimental details are shown in the corresponding figures, Table 1, and “Supplementary Information.” PTX (150 µg/kg b.w., i.p.) or PBS were injected 48 h before surgery.

**Table 1:** Summary of imaging experimental setup. The table shows the subdivisions of groups and time points. The number of animals used for statistics at every time point and animal group are displayed. Animals were injected with PTX 150 µg/kg b.w. i.p. or PBS 48 h before surgery. Images were obtained from mice in two main experimental settings. One main group was imaged during occlusion or sham surgery (Top). The other main group was examined at baseline (BL) and 48 h after occlusion/reperfusion (unilateral transient CCA) or sham surgery (96 h)

Group name	*n*	PTX	Surgery	Image time point
Sham PBS	5	-	Sham	0 h
Sham PTX	4	+	Sham	0 h
Occlusion PBS	6	-	Occlusion	0 h
Occlusion PTX	5	+	Occlusion	0 h
Sham PBS	5	-	Sham	BL / 96 h
Sham PTX	4	+	Sham	BL / 96 h
Occlusion/reperfusion PBS	5	-	Occlusion/reperfusion	BL / 96 h
Occlusion/reperfusion PTX	4	+	Occlusion/reperfusion	BL / 96 h

### Longitudinal Multiparametric MRI—Acquisitions and Analysis

Multiparametric MRI acquisitions were performed using a ClinScan 7-T small-animal MR scanner, a rat whole-body transmitting coil, and a 4-channel mouse brain surface receiving coil (Bruker Biospin). The imaging protocol consisted of anatomical T2-weighted images (T2WI), diffusion-weighted images (DWI), perfusion-weighted images (PWI), and multi-turbo-spin-echo T2-weighted acquisitions focusing specifically on the Bregma/Interaural 3.82 ± 0.25 mm brain region as previously performed [20–22] and detailed in the “Supplementary Information.”

### Volumes of Interest

All parametric images were coregistered to a common template using Pmod software (Bruker Biospin). Volumes of interest (VOIs) delimiting the striatal and cortical regions on both hemispheres were manually drawn at the above-described Bregma/Interaural brain regions using the T2WI as anatomical reference. The VOIs were overlaid on the PWI, ADC, and T2 maps followed by extraction of raw data. Lesion volumes were drawn on anatomical T2WI which covered the whole brain. Details are provided in the “Supplementary Information.”

### Single-Vessel Multi-gradient Echo (MGE) Imaging Experiment

We performed multi-gradient echo data acquisitions for single-vessel imaging using a 14.1 T / 26 cm magnet (Magnex, Oxford, UK) with an Avance III console (Bruker Biospin) and a 12-cm-diameter gradient providing 100G / cm with 150-µs rise time (Resonance Research, Massachusetts, U.S.A). A home-made RF surface coil (8-mm outer diameter) was used for single-vessel mapping. Details are provided in the “Supplementary Information.”

### Histology and Immunohistochemistry

For characterization of cerebral lesions, samples were stained with antibodies against CD31 (Abcam, Cambridge, UK), GFAP (Clone 6F2, Dako GmbH, Germany), HIF-1α (Clone ESEE 122, Abcam), and EPO (Clone H-162, Santa Cruz Biotechnology, Inc.). H&E staining was also performed. Details are provided in the “Supplementary Information.”

### Statistics

Sample size and power calculations were conducted and approved by the local animal ethical committees. Animal numbers for the main experiments are shown in Table 1. We evaluated non-Gaussian distribution for all experimental datasets previous to statistical testing using the Jarque–Bera test. Whole-body perfusion data presented several datasets that were non-normality distributed; therefore, statistical evaluation was performed using a 2-sided non-parametric Wilcoxon signed-rank test. All other experiments were evaluated using either 2-way or 3-way ANOVA [25], followed by multiple comparison corrections using Tukey’s honestly significant test [26]. Details are provided in the “Supplementary Information.”

## Results

### Differential Effects of a Functional Non-cerebral G_i_PCR KO on Blood Flow to Different Organs and Body Compartments

**Fig. 1:**
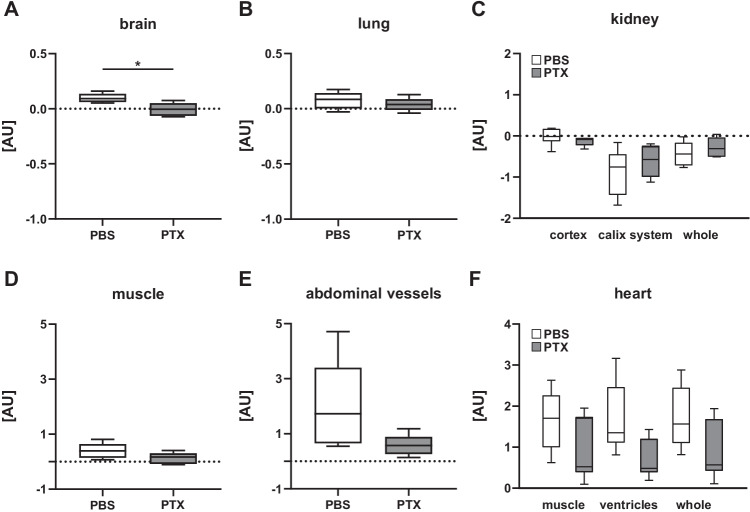
Functional non-cerebral G_i_PCR KO using PTX induces cerebral hypoperfusion. Whole-body perfusion was measured using dynamic contrast-enhanced imaging in PTX-pretreated and PBS-pretreated animals (*n* = 5 per group). **A** Wilcoxon matched-pairs signed-rank test found significant hypoperfusion in the brain of PTX-injected mice in comparison to PBS-treated animals (* *p* < 0.05). **B** Lung showed normal perfusion, whereas **C** kidney, **D** muscle, **E** abdominal vessels, and **F** heart yielded a hypoperfusion trend. Shown are median, 1st, and 3rd quartile of data distribution. The whiskers extend to the largest and smallest data point, respectively.

First, we examined the rough impact of PTX-induced functional non-cerebral G_i_PCR KO on blood flow in different organs and compartments using whole-body dynamic contrast-enhanced (DCE) MRI. ANOVA determined a statistically significant main group difference between PTX and control animals. To detect differences between the same organs of both groups, Wilcoxon matched-pairs signed-rank test was performed, and a statistically decreased blood flow was only found in the brain of PTX animals (Fig. 1A). As with some other organs and compartments, median blood flow in the ventricle, reflecting ejection fraction in this experimental setting, was lower than in the control group, but without being statistically significant (Fig. 1B–F). Blood flow to the renal cortex after PTX treatment also suggested sustained perfusion of the kidney (Fig. 1C). The contrast agent accumulated in the renal calyceal system of PTX-treated animals, also indicating continued tubular excretion. The systemic results led us to investigate the effects of PTX in the brain using MRI techniques with better spatial resolution.

### Reduction of Global CBF Following Functional Systemic Non-cerebral G_i_PCR KO with PTX

**Fig. 2:**
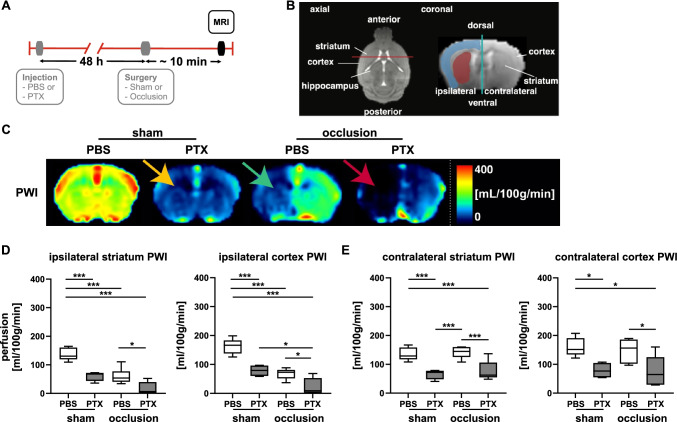
Functional PTX-induced non-cerebral G_i_PCR KO sensitizes for cerebral ischemia during permanent carotid artery occlusion. **A** Timeline of PBS/PTX injection, surgery (sham or CCA occlusion), and arterial spin-labeling MRI analysis. **B** Schematic overview of axial and coronal cross-sections of the mouse brain. The different brain regions of interest used for analysis are indicated. The red line shows the position of the cross-section corresponding to the coronal view. The blue line depicts the limit of ipsi (left)- and contra (right)-lateral brain hemispheres. **C** Perfusion-weighted images (PWI) indicate hypoperfusion of sham-operated PTX-treated mice (yellow arrow) compared to the sham PBS group. During left carotid artery ligation (occlusion), PBS-treated mice showed hypoperfusion visible in the ipsilateral hemisphere (green arrow), whereas PTX-pretreated mice exhibited global cerebral hypoperfusion, confirming the effects observed in whole-body perfusion analysis. Moreover, the perfusion of PTX-pretreated mice was interrupted in the ipsilateral hemisphere (red arrow) during occlusion, in comparison to animals receiving PBS. Shown are images of one representative mouse per group. Further examples are provided in Suppl. Fig. 1B. Corresponding quantification and statistics of CBF are shown in **D** for ipsi- and **E** for contralateral striatum and cortex (for details see Table 1). Statistical analysis was performed using 2-way ANOVA (* *p* < 0.05, *** *p* < 0.001). Shown are median, 1st, and 3rd quartile of data distribution. The whiskers extend to the largest and smallest data point, respectively.

To confirm and extend our observation of decreased CBF, we subjected the mice to an arterial spin labeling (ASL) MRI protocol (for details, see the “Materials and Methods” section and Fig. 2A, B), which shows the distribution of blood perfusion in the brain and provides reliable quantifications of CBF [21, 27, 28]. Coronal cross-sectional perfusion-weighted imaging (PWI) confirmed whole-brain hypoperfusion in PTX-pretreated sham-operated mice (Fig. 2C; yellow arrow). We quantified the CBF for the striatal and cortical regions (Fig. 2D, E). Both ipsi- and contralateral CBF were reduced by more than half in these regions compared to untreated sham-operated controls (Fig. 2D, E). Nevertheless, all of these reduced CBF values were above a range associated with ischemic lesions [29, 30]. Thus, in agreement with the DCE measurements (see Fig. 1), our ASL data clearly reveal a systemic suppressive effect of PTX on CBF.

### PTX Administration Sensitizes to Ischemia

Having established that a functional non-cerebral G_i_PCR KO with PTX had an effect in CBF per se, we examined the consequences of acute occlusion of one common carotid artery (CCA) in cerebral hypoperfusion (see the “Materials and Methods” section and Fig. 2A, B). As evident from PWI, unilateral CCA occlusion in control animals treated with PBS resulted in a large decrease on CBF ipsilateral to the occlusion (Fig. 2C, Suppl. Fig. 1B; green arrow). This is also reflected in the calculated CBF values, which showed a clear hypoperfusion for the ipsilateral striatum and cortex (Fig. 2D). However, the hypoperfusion did not reach a level described to cause ischemia and necrosis [29, 30]. Of note, blood flow in the contralateral regions remained stable (Fig. 2E), which should allow for potential compensatory blood flow to the hypoperfused regions [31].

In contrast, PTX-pretreated mice showed global cerebral hypoperfusion that was further aggravated ipsilateral to the unilateral CCA ligation resulting in a complete breakdown of perfusion in both the striatum and cortex (Fig. 2C, D; Suppl. Fig. 1B; red arrow). These ipsilateral values were below the threshold at which ischemic injury occurs [29, 30]. On the contralateral side, an extent of reduction occurred that we had already observed in the sham-operated mice pretreated with PTX, and that may impede compensatory blood flow to the hypoperfused ipsilateral regions (Fig. 2E, Suppl. Fig. 1B).

Our findings show that a functional non-cerebral G_i_PCR KO with PTX suppresses cerebral perfusion, which upon challenge by unilateral CCA occlusion severely disrupts CBF distal to the ligation, i.e., in the ipsilateral hemisphere. We were therefore interested in how perfusion subsequently developed and compared PWI at baseline and 48 h after surgery, which corresponded to 96 h after PTX administration (Suppl. Fig. 2A, B). The CBF in the brain of the PBS-injected mice, sham-operated or transiently CCA-occluded, was invariant from baseline post surgically at 48 h (Suppl. Fig. 2C-F). The corresponding CBF in the non-cerebral G_i_PCR KO mice was reduced albeit not significantly compared to baseline. Compared with the CBF of non-cerebral G_i_PCR KO mice during occlusion (see Suppl. Fig. 1B and Fig. 2D, E) the CBF of mice monitored 48 h later, i.e., 96 h after PTX dosing (see Suppl. Fig. 2C-F), indicated a partial recovery. However, there was no difference in CBF in PTX-pretreated mice regardless of whether they were sham-operated or transiently CCA-occluded 48 h before (see Suppl. Fig. 2C-F). This finding was in contrast to the different results in the two PTX-pretreated groups, i.e., sham-operated or transiently CCA-occluded at the time of occlusion (see Suppl. Fig. 1B and Fig. 2D, E). This prompted us to further investigate consequences of collapsed perfusion in non-cerebral G_i_PCR KO mice after transient unilateral CCA occlusion (Suppl. Fig. 3A).

### Functional Non-cerebral G_i_PCR KO Together with Transient Unilateral Carotid Artery Occlusion Leads to Cytotoxic and Vasogenic Edema

**Fig. 3:**
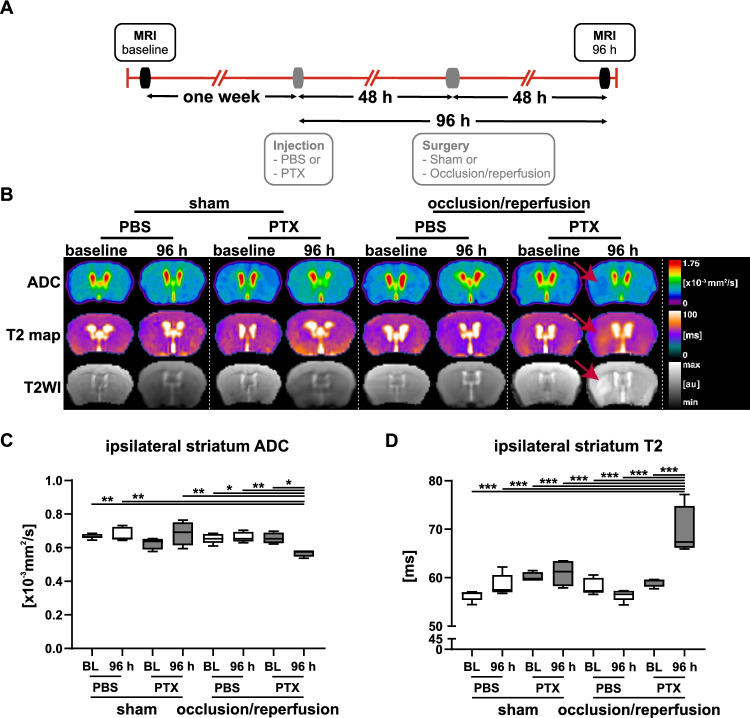
Cytotoxic and vasogenic edema in non-cerebral G_i_PCR KO following transient CCA occlusion. **A** Timeline of baseline MRI acquisition, PBS/PTX injection, surgery (sham or CCA occlusion), and post-operative MRI acquisitions. **B** Representative images of mouse brains showing the apparent diffusion coefficient (ADC), T2 map, and T2-weighted images (T2WI). Red arrows indicate the ischemic lesions in occluded PTX-pretreated mice consisting of reduced signal intensity of ADC images as well as hyperintensity in T2WI and T2 maps (for more details, see Tables 1, 2, and 3). Corresponding quantification and statistical analysis of ipsilateral ADC (**C**) and T2 (**D)** in the striatum. Only PTX-pretreated mice following transient CCA occlusion presented a lesioned striatum with increments in ADC, accompanied by an increased T2 relaxation time. Statistical analysis was performed using 3-way ANOVA (* *p* < 0.05, ** *p* < 0.01, *** *p* < 0.001). Shown are median, 1st, and 3rd quartile of data distribution. The whiskers extend to the largest and smallest data point respectively.

Diffusion-weighted images (DWI) provide a measurement of diffusion that can be quantified in the apparent diffusion coefficient (ADC) using MRI. ADC restrictions in the brain are the gold standard to identify ischemic stroke lesions, which have been shown to strongly correlate to final infarct lesions in tissue sections [32–35]. Diffusion restrictions have been known to start rapidly after stroke onset, peaking within one day, followed by slow value normalization [36, 37]. Consistent with these previous reports, PTX-pretreated and occluded mice already showed incipient ADC restrictions during occlusion (see Suppl. Fig. 3B-D), which were still evident in the mice imaged at 48 h post-surgery, corresponding to 96 h after PTX administration (Fig. 3B; red arrow, Fig. 3C). These ADC restrictions were clearly demarcated in DWIs of these mice (Suppl. Fig. 3B-D).

**Table 2:** Contralateral striatum results

Measurement (unit)	Group	BL	0 h	96 h
ADC (× 10^–3^ mm^2^/s)	Sham PBS	0.66 ± 0.04	0.68 ± 0.15	0.68 ± 0.13
	Sham PTX	0.64 ± 0.08	0.62 ± 0.26	0.66 ± 0.15
	Occlusion PBS	0.65 ± 0.08	0.55 ± 0.13	0.65 ± 0.11
	Occlusion PTX	0.65 ± 0.08	0.62 ± 0.15	0.64 ± 0.08
T2 relaxation time (ms)	Sham PBS	56.7 ± 2.0	59.0 ± 3.4	58.7 ± 11.1
	Sham PTX	59.2 ± 2.4	55.6 ± 7.8	60.6 ± 5.5
	Occlusion PBS	61.0 ± 5.6	53.8 ± 3.6	56.8 ± 2.6
	Occlusion PTX	58.5 ± 3.8	56.2 ± 3.0	56.4 ± 2.4

**Table 3:** Contralateral cortex results

Measurement (unit)	Group	BL	0 h	96 h
ADC (× 10^–3^ mm^2^/s)	Sham PBS	0.65 ± 0.12	0.72 ± 0.20	0.65 ± 0.12
	Sham PTX	0.65 ± 0.15	0.65 ± 0.08	0.68 ± 0.15
	Occlusion PBS	0.66 ± 0.04	0.58 ± 0.23	0.67 ± 0.04
	Occlusion PTX	0.60 ± 0.06	0.60 ± 0.13	0.67 ± 0.06
T2 relaxation time (ms)	Sham PBS	59.8 ± 7.1	58.4 ± 4.0	59.9 ± 7.1
	Sham PTX	57.9 ± 4.7	57.8 ± 4.4	61.9 ± 4.7
	Occlusion PBS	59.6 ± 3.9	56.4 ± 4.4	59.6 ± 3.9
	Occlusion PTX	60.8 ± 1.6	57.0 ± 1.4	57.1 ± 1.6

Moreover, T2 relaxation maps and T2WI representing vasogenic edema, demonstrated hyperintense signals only in the PTX-pretreated occluded animals at the latest timepoint (Fig. 3B, D; Suppl. Fig. 3B, E, F), consistent with previous literature [36, 37]. As the occluded animals pretreated with PTX showed vasogenic edema, we quantified edema volume in relation to their anatomical structures (Suppl. Fig. 4). In contrast, no signs of cytotoxic or vasogenic edema were detectable in both sham-operated groups and the PBS-treated occluded group 48 h after occlusion (Figs. 3C, D).

**Fig. 4:**
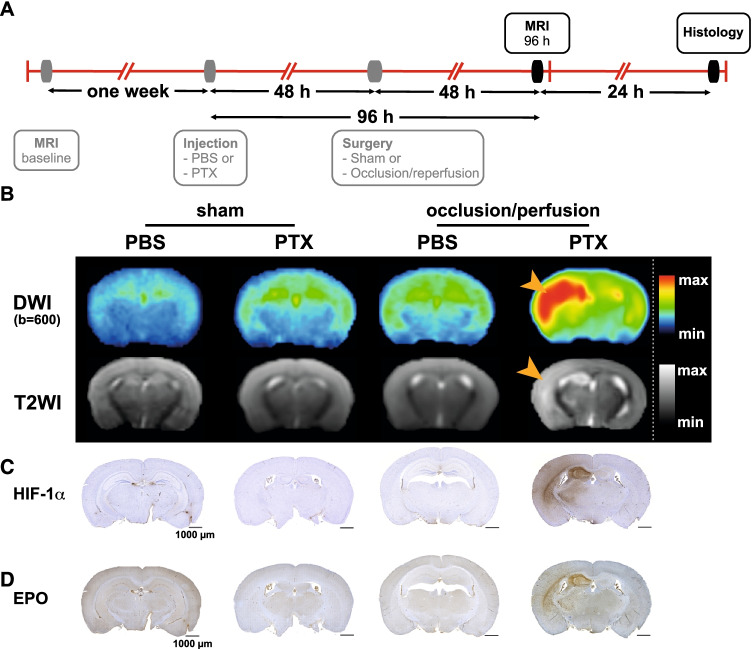
Colocalization of DWIs and T2WIs with immunohistochemical ischemia in occluded PTX-pretreated mice. **A** Timeline of PBS/PTX injection protocol, surgery, and MRI acquisition. **B** DWI (*b* value = 600 s/mm^2^) and T2WI of animals at 96 h on the coronal projection. The occluded PTX-pretreated mice show hyperintensities in the striatal, hippocampal, and cortical brain regions on DWI and T2WI (orange arrowheads). Animals of the other groups showed no visible lesions. For more details, see Table 1. **C** HIF-1α is stained in hippocampal stroke regions and marks the infarcted region colocalizing with the DWIs. **D.** Staining of the hypoxia-inducible cytokine EPO shows a focalized lesion similar to the HIF-1α-positive hypoxic region further confirming an ischemic event. Immunohistochemistry was done in *n* = 4 mice per group.

Because cytotoxic and vasogenic edema developed only in PTX-pretreated animals with transient CCA occlusion, we performed histological and immunohistochemical analyses to confirm the presence of an ischemic stroke phenotype, as we have previously done in other stroke models [20, 35]. Detection of ischemic lesions using hypoxia-inducible factor 1α (HIF-1α) and erythropoietin (EPO) immunohistochemistry has been previously shown to clearly delimit the infarct core and the peri-infarct stroke region [38, 39] (Fig. 4A-D; Suppl. Fig. 5). The immunohistochemical staining showed focal lesions in the PTX-pretreated and CCA-occluded animals demonstrating ischemia ipsilateral to the occlusion, which perfectly colocalized with hyperintense lesions seen in DWIs and T2WIs (Fig. 4). Furthermore, H&E staining and immunohistochemistry for the endothelial markers CD31 and GFAP (Suppl. Fig. 5) revealed prominent lesions with neuronal pallor, vacuolation of the neuropil and edema (H&E) in various regions of the ipsilateral hemisphere, as well as blood vessels (CD31) and reactive gliosis (GFAP). Thus, clear signs of ischemic stroke through *in vivo* imaging were confirmed in PTX-pretreated transiently CCA-occluded animals using immunohistochemistry and histology.

### Functional Non-cerebral G_i_PCR KO with PTX Reduces Patency of Individual Cortex-Penetrating Microvessels

**Fig. 5:**
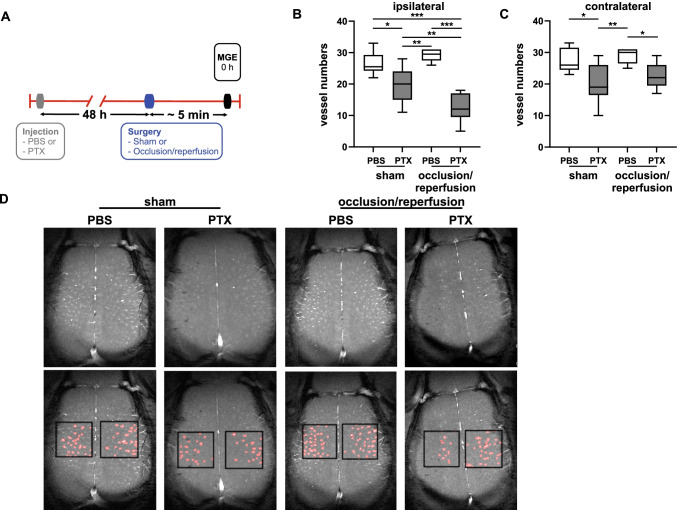
Functional non-cerebral G_i_PCR KO reduces patency of cortex-penetrating microvessels. **A** Timeline of PBS/PTX injection and surgery protocol following multi-gradient echo (MGE) MRI acquisition. These experiments were performed during occlusion or sham surgery. Results of quantification of vessel numbers in the ipsi- (**B)** and (**C**) contralateral cortex (*n* = 6–9). Vessel numbers of PTX-pretreated mice are reduced in both hemispheres, which is further aggravated upon occlusion in the ipsilateral cortex. Statistical analysis was performed using 2-way ANOVA (* *p* < 0.05, ** *p* < 0.01, *** *p* < 0.001). **D** Representative pictures from all four groups measured by MGE (upper panel). The black boxes mark the assessed areas, and the red dots are the identified vessels (lower panel). Shown are median, 1st, and 3rd quartile of data distribution. The whiskers extend to the largest and smallest data point, respectively.

We investigated whether hypoperfusion was associated with collapsed microvessels. To specifically investigate the immediate response of microvessels to CCA occlusion, we used a multi-gradient echo (MGE) MRI sequence (Fig. 5A) [40–42]. High-resolution MGE-MRI provides a penetrating microvessel-specific measurement of the cortex that allows the estimation of microvascular collapse. Comparison of PBS-treated mice regardless of CCA occlusion revealed no difference in the number of vessels in both hemispheres (Fig. 5), indicating a normal microvascular function.

In contrast, the PTX-induced functional non-cerebral G_i_PCR KO provoked a reduction of quantifiable microvessels in the cortex of both hemispheres compared to the PBS groups (Fig. 5). The effect was further aggravated in the PTX-pretreated occluded mice, where an even more prominent number of microvessels collapsed in the ipsilateral cortex (Fig. 5). In combination with our perfusion experiments, these data suggest that PTX does not only cause global cerebral hypoperfusion but also micro-cerebrovascular collapse, which has also been described to occur under low-perfusion pressure in heart vessels [7].

## Discussion

Cerebrovascular functions of G_i_PCR-driven signaling are still largely unknown. To gain more insight, we employed the highly specific inhibitor PTX in order to specifically disrupt extraneuronal G_i_PCR signaling. Our results point to previously unrecognized functions of G_i_PCR signaling in the regulation of CBF and possibly systemic blood flow. Furthermore, extraneuronal functional PTX-induced non-cerebral G_i_PCR KO in combination with unilateral CCA produces brain lesions with similar imaging characteristics to human ischemic stroke.

One major drawback of the functional non-cerebral G_i_PCR KO with PTX is the ubiquitous nature of the KO in a multitude of systemic cellular processes. The systemic non-cerebral G_i_PCR KO may induce alterations in various systems, such as cardiovascular and immune system. In fact, it is used to establish the pertussis toxin-induced reversible encephalopathy dependent on monocyte chemoattractant protein-1 overexpression (PREMO) model, consisting on the injection of *Mycobacterium tuberculosis* and two injections of PTX [43]. We have previously shown that although PTX-sensitive G_i_ proteins are ubiquitously expressed, a single extraneuronal application of the toxin *in vivo* does not modify neuronal G_i_PCR and does not cross through the intact BBB [19]. Therefore, it is possible under this specific setting, to evaluate the effects of PTX in the perfusion of brain vessels and in systemic perfusion, without unwanted effects in neurons. We evaluated systemic hemodynamic effects using whole-body perfusion in order to reveal major possible alterations, and although we found only significant effects in the brain, other effects on systemic hemodynamics cannot be excluded. In fact, it has been demonstrated that PTX induces changes in blood pressure in hypertensive rats [2]. Moreover, it has been reported that in the cardiovascular system, PTX activity induces vessel size-dependent changes in vascular resistance [7], impairs endothelial Ca^2+^ influx [8], or lowers Ca^2+^ sensitivity of vasoconstriction in response to noradrenaline [2]. In line with these previous works, our findings now reveal a relevant effect in global cerebral hypoperfusion and microvascular collapse of cerebral vessels. The microvessel dysfunction could be at least partially mediated by interference with vascular G_i_ protein-mediated signaling affecting nitric oxide, β-adrenergic, angiotensin II type 1, serotonin-1A, or relaxin receptor function [2, 15, 18, 44]. Moreover, PTX has been shown to inhibit endothelium-dependent relaxation in hypercholesterolemic and atherosclerotic arteries [15, 16], which specifically links a disrupted G protein-mediated transduction to microvascular dysfunction. Indeed, chronic hypertension, dyslipidemias, diabetes, and increased age have been correlated to hypoperfusion and microarterial impairment [45–47].

Interestingly, PTX has been recently reported to be neuroprotective due to a reduction of glutamate-induced calcium influx into ischemic neurons [48]. Tang et al*.* injected PTX as a neuroprotectant at a dose of 40 µg/kg b.w. 30 min after applying a permanent middle cerebral artery occlusion. This occlusion triggered a BBB breakdown, allowing PTX to enter the brain [37, 49]. Consequently, Tang et al*.* injected PTX at a lower dose and at a time when the ischemic brain had a permeable BBB and could potentially benefit from inhibition of calcium influx. However, no perfusion deficits were observed in this study. In the current study, we administered the toxin again at 150 µg/kg b.w. 48 h before carotid artery occlusion; thus, the BBB was intact at the moment of PTX injection and not able to reach the neurons [19]. The comparison of the work from Tang et al*.* to our study is an excellent reminder of how timing and dosage of therapeutic interventions, especially in niche compartments, are important for outcome.

The significance of our findings in the clinical field is directly related to the involvement of G protein signaling alterations in the pathogenesis of neurodegenerative and cerebrovascular diseases. G protein signaling is involved with neurotransmitters such acetylcholine, GABA (gamma-aminobutyric acid), and glutamate. Here, for example, the acetylcholine receptor has been associated to formation of Aß peptide and neurofibrillary tangles in Alzheimer’s disease [50]. From a vascular perspective, alterations in G protein signaling involving monoamines such as adrenaline, noradrenaline, serotonin, dopamine, and histamine could be directly associated with cerebral hypoperfusion, a well-known imaging hallmark of neurodegenerative diseases [21, 51, 52]. Cerebral hypoperfusion is also a common risk factor in cerebrovascular diseases such as cerebral microbleeds and stroke [53, 54]. Therefore, G_i_PCR-driven signaling for the maintenance of CBF may be relevant to identify novel therapeutic targets. The PTX-triggered CBF impairment sensitized the brain to ischemic injury by disabling the mechanisms of blood flow regulation, an interesting effect that requires further mechanistic clarification focusing on the deficiency of specific G protein isoforms. The impaired hemodynamic stability and responsiveness of the cerebrovascular system caused by functional non-cerebral G_i_PCR KO in mice are reminiscent of observed hypoperfusion and vascular dysfunction in humans with chronic vascular disease, which is also predictive of human stroke severity [47].

Up to now, blocked GiPCR signaling had not yet been linked to the occurrence of cerebrovascular hypoperfusion and vascular collapse. It will be interesting to identify the specific G_i_PCRs involved in the maintenance of CBF and vascular tone. Furthermore, the effects of hypoperfusion and microvascular collapse induced by functional non-cerebral G_i_PCR KO may be useful for neuroscience, functional neuroimaging, and neurooncology.

## Supplementary Information

Below is the link to the electronic supplementary material.

Supplementary file1 (PDF 3.04 mb)
