# Supplementary material for: Cerebrovascular Gi Proteins Protect Against Brain Hypoperfusion and Collateral Failure in Cerebral Ischemia
Source: Mol Imaging Biol. 2022 Sep 8;25(2):363–74. doi: 10.1007/s11307-022-01764-8 (PMC10006265; doi:10.1007/s11307-022-01764-8)
Supplement: Supplementary file 1 — Supplementary file1 (PDF 3.04 mb) [file 11307_2022_1764_MOESM1_ESM.pdf]

Supplementary Material

Anesthesia and pain management

Surgical procedures were performed on a warm pad under isoflurane anesthesia at 4% induction vaporized in 100% O_2 at a flow rate of 1.5 L/min, followed by 1–2% of isoflurane for maintenance. To provide analgesia Metamizole (100 mg/kg b.w., i.p.) was injected 10 min before the surgeries followed by Carprofen treatment (4 mg/kg b.w., subcutaneously per day) for pain management. For imaging experiments animals were anaesthetized using isoflurane (induction at 4% and maintenance at 1-2% vaporized in 100% O_2 at a flow rate of 1.5 L/min) and placed on a MRI-compatible bed with an integrated water heating system (Bruker Biospin). Body temperature was monitored using a rectal probe and maintained at 37.0 ± 0.5 °C. The head was secured on the brain-dedicated bed using stereotactic pins. Breathing rate was monitored during the MRI acquisitions and used for image acquisition gating.

Whole body semi-quantitative perfusion estimations

In order to determine whether PTX produced systemic perfusion changes, mice weighing 20-23 g were evaluated using dynamic contrast enhanced (DCE) MRI. PTX or PBS was injected i.p. (150 µg/kg b.w.) into 5 mice per group. Dynamic contrast enhanced (DCE) images were acquired 48 h after PTX injection using a Biospec 7T small-animal MRI scanner (Bruker Biospin, Ettlingen, Germany) with a whole-body transmit-receive volume coil with 40 mm inner diameter which covered the whole body of the mouse. For anatomical orientation, a T2-weighted Turbo Rapid Acquisition with Relaxation Enhancement (RARE) sequence was acquired with the following parameters: time of repetition (TR) = 1257 ms, time of echo (TE) = 25 ms, field of view (FOV) = 40 mm × 40 mm, matrix size = 256 × 256, slice thickness (ST) = 1 mm, repetition = 1. For DCE-MRI, the routinely used low molecular weight (0.604 kDa) contrast agent Gadobutrol (Gadovist® 1 mmol/ml, Bayer Schering Pharma, Berlin, Germany) was diluted to a concentration of 0.2 mmol/ml and i.v.-injected through a tail vein to both

control and PTX mice. A dynamic T1-weighted sequence was acquired by applying multiple coronal slices covering the whole body transversally in multiple organ regions with the following parameters: TR = 45 ms TE = 2.1 ms, flip angle = 20°, FOV = 52 mm × 26 mm, matrix size = 128 × 64, ST = 1 mm, repetitions = 60. The DCE scan started approximately 25 s before the i.v. contrast injection and was acquired for 5 min. DCE post-processing was performed using an in-house developed Matlab script adapted from Khalifa et al.[1]. Briefly, the time-signal intensity curve was fitted with a linear regression from the injection time to 30 s post-injection; a voxel-wise semi-quantitative parametric map was generated. Data extraction from the maps was generated by manually defining VOIs in the brain, heart, lungs, paravertebral muscles, abdominal vessels, and kidneys.

Surgical interventions – occlusion, transient occlusion/reperfusion, and sham surgery

The vessel occlusion surgery of animals of the PTX-pretreated and PBS-pretreated occlusion groups consisted of the ligation of the left common carotid artery (CCA) (timeline see Fig. 2A). These animals were dissected under a surgical microscope using a small median incision above the trachea, through the sub-maxillary salivary glands exposing the CCA. The CCA was dissected carefully avoiding the *vagus* nerve. The CCA was exposed and was tightly ligated using a non-absorbable suture. The surgeon visually confirmed complete flow disruption following the ligation. An approximate total surgery time of 15 min per animal was used for each surgery, where ligation of the CCA occurred at approximately 12 min. Upon closing the wound, the animals were immediately transported and scanned in the MRI, hence the scan occurs under complete occlusion of one common carotid artery.

Animals belonging to the PTX-pretreated occluded and PBS-treated occluded groups, which were scanned at baseline and 48 h post-surgery, were submitted to a transient occlusion of the CCA (timeline see Fig. 3A). The occlusion was performed using a removable vascular micro clamp (Fine Science Tools, Heidelberg, Germany) exerting an approximate pressure of 85 g/mm^2 for 30 min on one CCA. The surgeon visually confirmed complete flow disruption

and subsequent reperfusion of the CCA following clamp removal after 30 min. Taking into account the opening and closing of the surgical wound, each procedure lasted approximately 45 min. No MRI was performed at the moment of occlusion. The mice were allowed to recover in the home cages and were scanned 48 h post-surgery.

The PBS-treated sham-operated and PTX-pretreated sham-operated groups underwent a "sham" surgical protocol that consisted of the same surgical procedure as described above. However, instead of an occlusion, only a dissection of the CCA was performed with subsequent closing of the surgical wound with a similar surgical time in order to replicate surgical metabolism and anesthesia effects.

Longitudinal multiparametric MRI evaluations

Multiparametric MRI acquisitions were performed using a Clinscan 7 T small-animal MR scanner, a rat whole-body transmitting-coil, and a 4-channel mouse brain surface receiving-coil (Bruker Biospin). T2WI were performed using a 3D-spoiled turbo spin echo sequence (256 x 161 matrix, 35 x 57 mm^2 field of view (FOV), repetition time (TR) = 3000 ms, echo time (TE) = 205 ms, slice thickness (ST) = 0.21 mm). DWI were acquired using a one directional Half-Fourier Acquisition Single-shot Turbo Spin-Echo (HASTE) sequence on the coronal plane covering most of the brain (192 x 120 matrix, 25 x 40 mm^2 FOV, TR = 5000 ms, TE = 112 ms, flip angle = 180°, number of excitations = 4, ST = 1 mm, b-values = [0, 150, 250, 400, 600, 800, 1000] s/mm^2). DWI with b-values: [150, 250, 400, 600, 800 and 1000] s/mm^2 were used for the ADC map calculation. Additionally, a multi-echo spin-echo T2-weighted sequence was acquired for calculation of a T2 Map covering the whole brain (128 x 96 matrix, 25 x 34 mm^2 FOV, TR = 5000 ms, 12 echo times = 15-180 ms, flip angle = 180°, ST = 1 mm). T2 maps were calculated using all acquired echo times = 15-180 ms. PWI were acquired using an arterial spin labelling (ASL) sequence as we have previously described [2, 3]. One slice of coronal plane Flow-sensitive Alternating Inversion Recovery and a True Fast Imaging with

Steady Precession (FAIR True-FISP) acquisition protocol (64 x 64 matrix, 25 x 25 mm^2 FOV, TR = 4.1 ms, TE = 2.05 ms, inversion time = 1800 ms, interscan time = 7000 ms, flip angle = 70°, number of inversions = 30, ST = 1.2 mm) was positioned within the following brain coordinates: Bregma/Interaural 3.82 ± 0.25 mm. CBF from ASL derived PWI using a self-developed Matlab script (The MathWorks Inc) and a partition coefficient of $\lambda = 0.9$ as described [2]. Apparent diffusion coefficient (ADC) and T2-relaxation images (T2 maps) were calculated using the Syngo console (Siemens Healthcare, Erlangen, Germany).

Calculation of Lesion volume

Lesion volumes were manually drawn on the anatomical whole brain T2WI at 48 h post occlusion. We further measured the complete volume of the ipsilateral striatal, cortical and hippocampal regions. This allowed us to calculate the percentage of lesion per ipsilateral brain region for each mouse.

Single vessel multi-gradient echo (MGE) imaging experiment

To detect individual arterioles and venules, a 2D multi-gradient echo sequence was used with the following parameters: TR: 50 ms, TE: 2.5, 5, 7.5, 10, 12.5, 15 ms; flip angle: 40°, matrix: 192 x 192, in-plane resolution: 50 x 50 μm^2, 500 μm thickness for 14T. The single vessel map was only based on the first TE to examine the number of bright dots from both arterioles and venules due to in-flow effect. Individual vessel voxels were determined based on their higher signal intensity than the mean signal intensity plus 2-times standard deviation of the local area in a 5 x 5 kernel [4–6]. Animal groups were the same as in the main multiparametric imaging experiment.

Histology and Immunohistochemistry

For histology 3-5 μm-thick sections were cut and stained with hematoxylin and eosin (H&E). Immunohistochemistry was performed on an automated immunostainer (Ventana Medical

Systems, Inc., Tucson, Arizona, USA) according to the company's protocols for open procedures with slight modifications. Appropriate positive and negative controls were used to confirm the adequacy of the staining.

Statistical analysis

We evaluated non-gaussian distribution for all experimental datasets previous to statistical testing using the Jarque Bera test. The whole-body perfusion experiments yielded several non-normally distributed datasets; therefore, we separately evaluated all those data using the non-parametric Wilcoxon signed-rank test. In the single timepoint imaging experiments (during occlusion, Fig. 2 and Fig. 5), 2-way ANOVA was used to evaluate main effects of surgery (Occlusion or Sham) and therapy (PTX or PBS). 3-way ANOVA was used to evaluate the experiments with two time points (Fig. 3), in addition to main effects of surgery (Occlusion or Sham), and therapy (PTX or PBS). Every statistical test was independently performed per brain region. An alpha level of 0.05 and a confidence interval of 95% were considered significant. All statistical analyses were performed using Matlab's Statistical Tool Box (The Math Works Inc.) or GraphPad Prism (GraphPad Prism Inc.; Version 9). The numbers of animals used per group and time point for statistical analysis are shown in Table 1. Supplementary tables 1-3 depict the statistical data in more detail. The results presented in boxplots are interpreted as follows: The middle line represents the calculated median of the data, the upper border of the box represents the 1st quartile of data distribution, the bottom border represents the 3rd quartile and the whiskers show the outmost data points. In the text and tables, results are presented as median and range of the data.

References

1. Khalifa F, Soliman A, El-Baz A, et al (2014) Models and methods for analyzing DCE-MRI: A review. Med Phys 41:124301. doi: 10.1118/1.4898202

2. Castaneda Vega S, Weinl C, Calaminus C, et al (2017) Characterization of a novel murine model for spontaneous hemorrhagic stroke using in vivo PET and MR multiparametric imaging. Neuroimage 155:245–256. doi: 10.1016/j.neuroimage.2017.04.071

3. Maier FC, Wehrl HF, Schmid AM, et al (2014) Longitudinal PET-MRI reveals β-amyloid deposition and rCBF dynamics and connects vascular amyloidosis to quantitative loss of perfusion. Nat Med 20:1485–1492. doi: 10.1038/nm.3734

4. Yu X, He Y, Wang M, et al (2016) Sensory and optogenetically driven single-vessel fMRI. Nat Methods 13:337–340. doi: 10.1038/nmeth.3765

5. He Y, Wang M, Chen X, et al (2018) Ultra-Slow Single-Vessel BOLD and CBV-Based fMRI Spatiotemporal Dynamics and Their Correlation with Neuronal Intracellular Calcium Signals. Neuron 97:925-939.e5. doi: 10.1016/j.neuron.2018.01.025

6. Qian C, Yu X, Pothayee N, et al (2014) Live nephron imaging by MRI. Am J Physiol Physiol 307:F1162–F1168. doi: 10.1152/ajprenal.00326.2014

Supplementary Figures

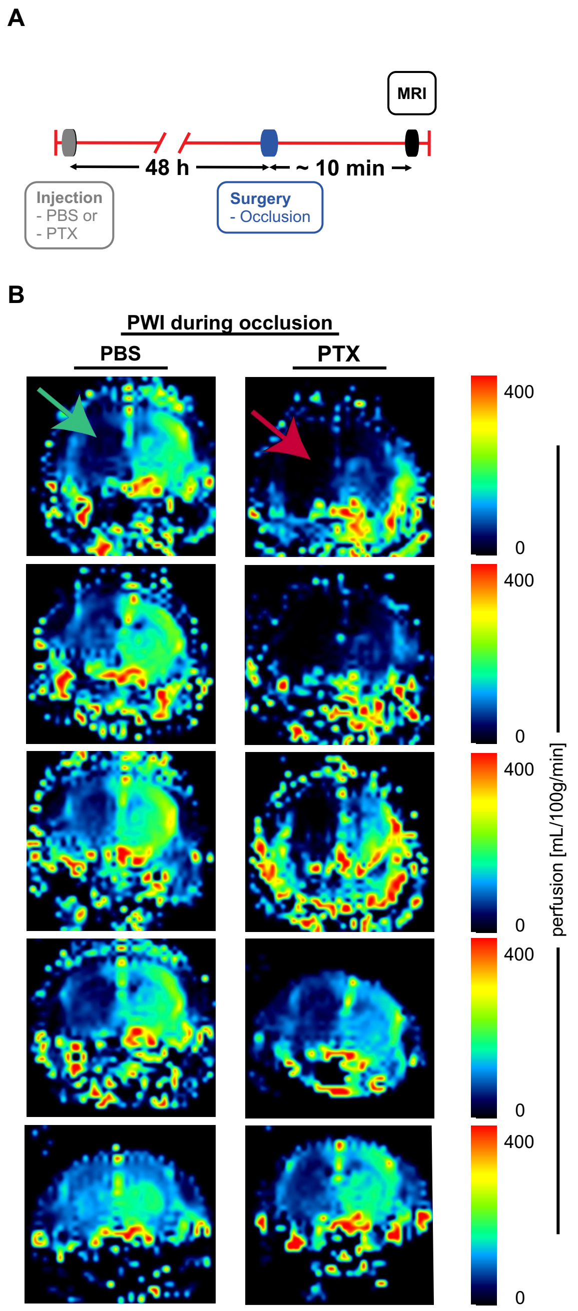

Suppl. Fig. 1. Perfusion during unilateral carotid artery occlusion. A. Timeline of PBS/PTX injection, unilateral common carotid artery occlusion surgery and arterial spin-labeling MRI perfusion analysis. **B.** The coronal images on the left column are the cerebral blood flow images of the PBS-pretreated occlusion group. There is a clear reduction in perfusion on the ipsilateral hemisphere (green arrow) as compared to the contralateral right hemisphere. The images on the right column correspond to PTX-pretreated animals during occlusion. CBF is severely disrupted in the ipsilateral brain in these animals and strongly reduced on the contralateral hemisphere, as compared to the PBS-injected mice.

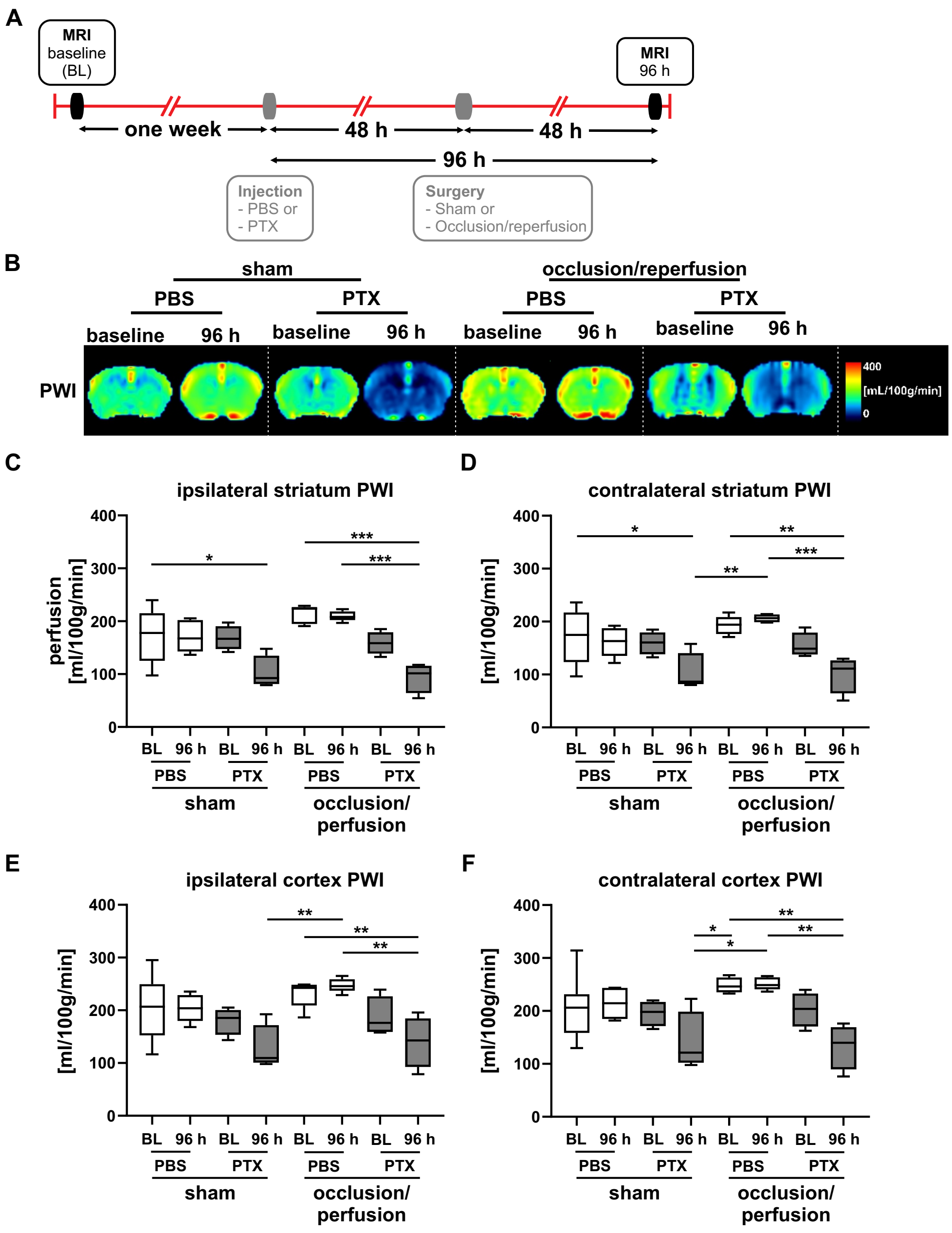

Suppl. Fig. 2. Longitudinal recovery of cerebral perfusion following transient CCA occlusion in PTX-pretreated mice. A. Timeline of baseline (BL) MRI analysis, PBS/PTX injection protocol, transient common carotid artery occlusion surgery, followed by post-surgery MRI analysis. Baseline defines the analysis performed one week pre-surgery. **B.** Shown are representative coronal perfusion weighted images (PWI) at baseline and 96 h after PBS/PTX-pretreatment. PTX-injected animals with and without transient occlusion, presented global brain hypoperfusion in comparison to baseline. PBS-injected animals, with or without transient occlusion, presented no hypoperfusion at 96 h. **C-F.** Corresponding quantification and statistical analysis of ipsi- and contralateral striatum and cortex perfusion rates at baseline and 96 h. Statistical analysis was performed using 2-way ANOVA (* $p < 0.05$, ** $p < 0.01$, *** $p < 0.001$). Shown are median, 1st and 3rd quartile of data distribution. The whiskers extend to the largest and smallest data point respectively.

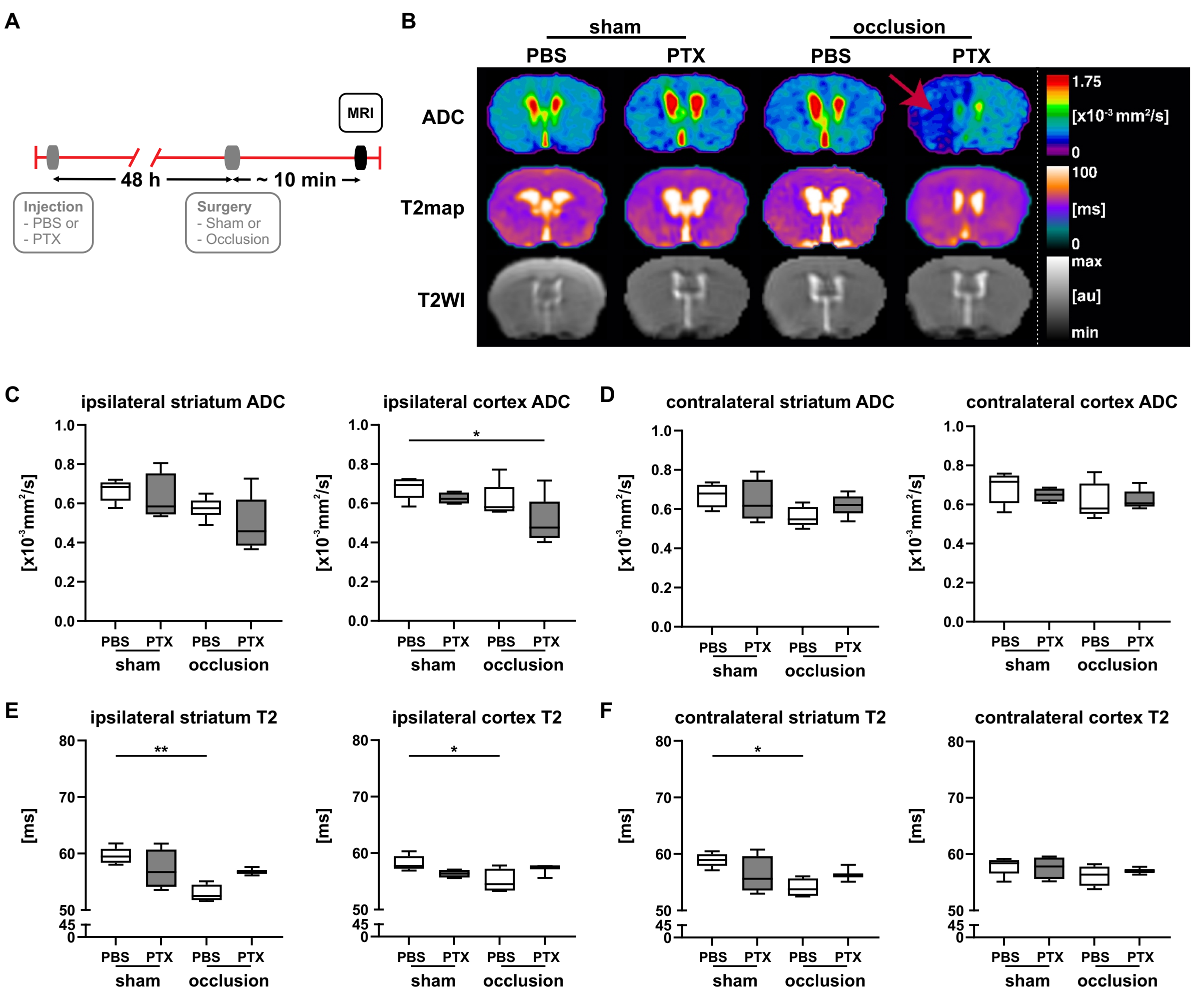

Suppl. Fig. 3. Functional G$_i$PCR KO induces cytotoxic edema during CCA occlusion. A. Timeline of PBS/PTX injection, surgery (sham or CCA occlusion) and MRI acquisition during occlusion. **B.** Representative apparent diffusion coefficient (ADC), T2 map and T2-weighted images (T2WI) for each animal group. Occluded PTX-pretreated mice presented remarkable reductions in ADC after merely 20 to 30 min after carotid artery occlusion, capturing the early onset of cytotoxic edematous changes corresponding to stroke lesions. Statistical analysis of ipsilateral (**C**) and contralateral (**D**) ADC in striatum and cortex as well as ipsilateral (**E**) and contralateral (**F**) T2 in striatum and cortex. Statistical analysis was performed using 2-way ANOVA (* $p < 0.05$, ** $p < 0.01$). Shown are median, 1st and 3rd quartile of data distribution. The whiskers extend to the largest and smallest data point, respectively. For more details see Table 1-3.

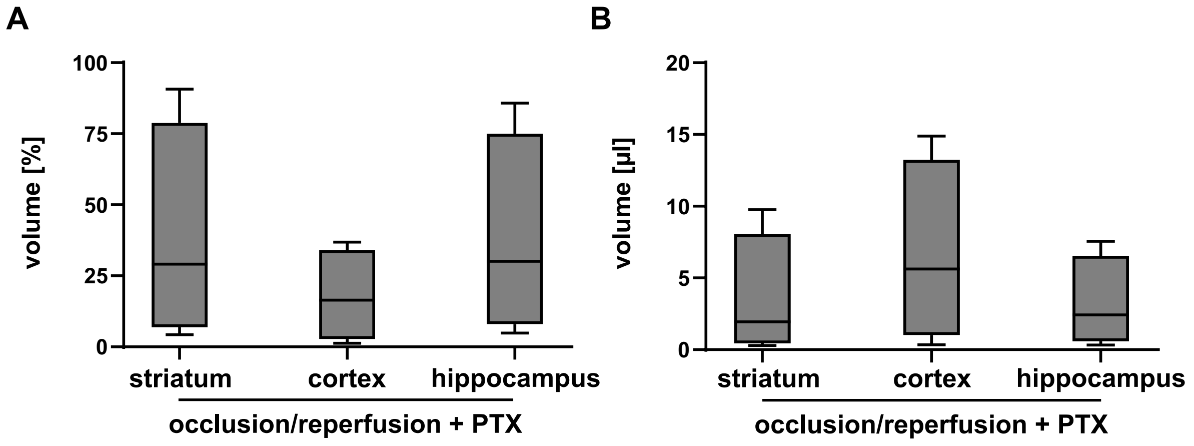

Suppl. Fig. 4. Lesions at 96 h were detectable in striatum, cortex and hippocampus of PTX-pretreated mice. A. Stroke lesions were identified in T2 weighted images, mostly distributed ipsilateral to the occlusion in the striatal, cortical, hippocampal regions, and quantified as described in the methods section. The median percentage of the respective lesion per region is shown. **B.** Median stroke volumes in the indicated brain regions. Shown are median, 1st and 3rd quartile of data distribution. The whiskers extend to the largest and smallest data point respectively.

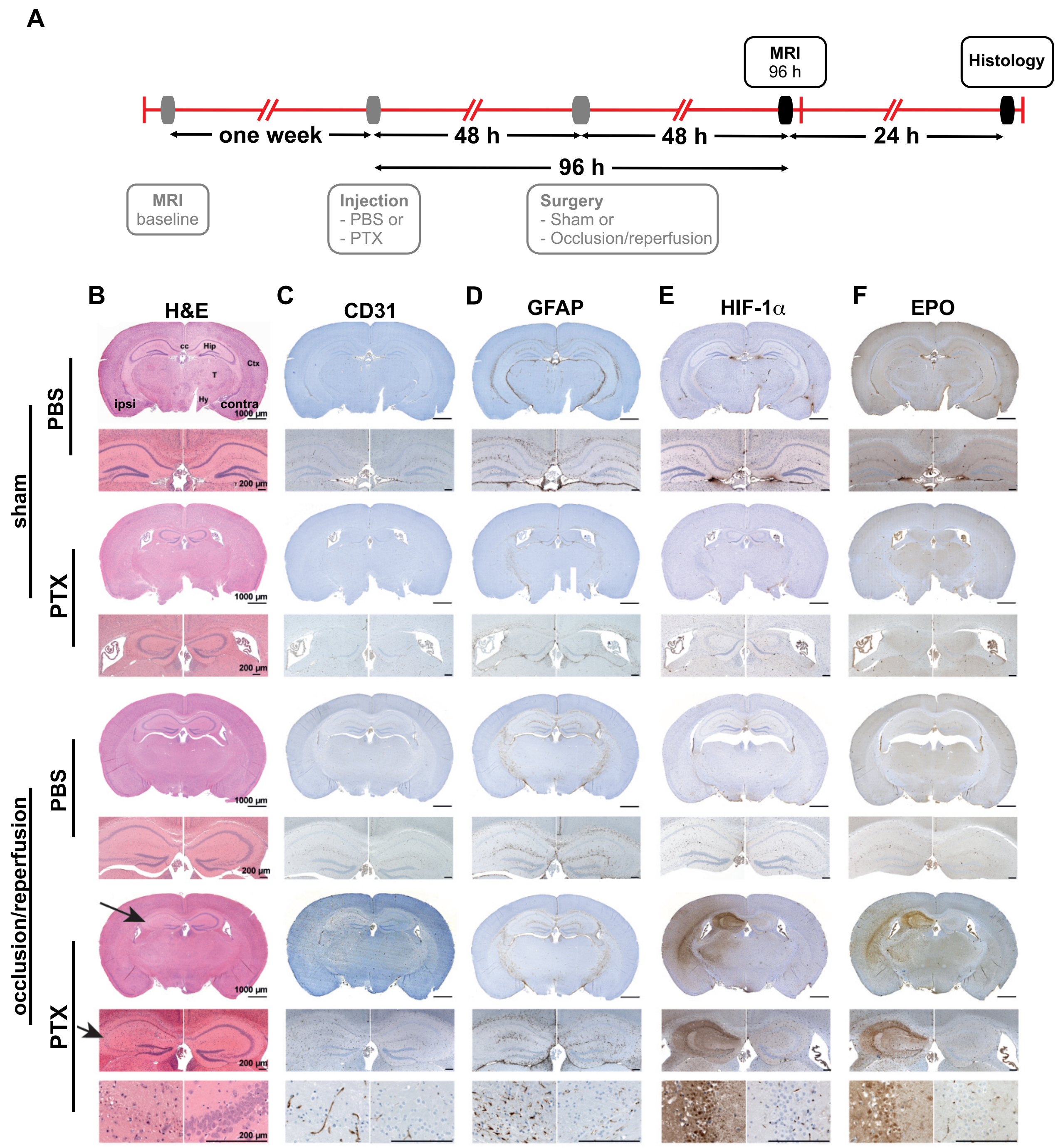

Suppl. Fig. 5. PTX-treatment combined with carotid artery occlusion triggers severe histopathological ischemic stroke lesions. Representative histological slides correspond to the brain images shown in Fig. 4. **A.** Timeline of experiment acquisitions. **B.** H&E staining of brain sections from occluded PTX-pretreated mice shows edema and erasing of CA1 and CA3 hippocampal regions (black arrows) and edema in the striatal region. **C.** Strong expression of the angiogenesis marker CD31 can be observed in the stroke area. **D.** GFAP visualizing activated glial cells is expressed in the ischemic region. **E.** HIF-1α is stained in hippocampal stroke regions and marks the infarcted region colocalizing with the DWIs. **F.** Staining of the hypoxia-inducible cytokine EPO shows a focalized lesion similar to the HIF-1α-positive hypoxic region further confirming an ischemic event. CC: Corpus Callosum, Hip: Hippocampus, CTX: Cortex, T: Thalamus, Hy: Hypothalamus.

Supplementary Table 1. Wilcoxon signed-rank test statistics for whole body perfusion measurements. The table shows value of the Ranksum statistic (Rank), the result of z-statistic (z-value), and the corresponding p-value.

Organ	Rank	Z-value	p-value
Brain	38	2.09	0.036
Lung	31	0.62	0.531
Whole kidney	24	-0.62	0.530
Muscle	34	1.25	0.210
Vessels	36	1.67	0.095
Whole heart	34	1.25	0.210

Supplementary Table 2. Multivariate analysis of variance (ANOVA) for perfusion, ADC, and T2 measurements. The table shows the details of analyses of variance performed on the multiple datasets and the results of the analyses (2-way ANOVA in grey, 3-way ANOVA in white). The variables considered for 2-way ANOVA were surgery type (Sham or Occlusion) and treatment (PTX or PBS). For 3-way ANOVA, time was added as a variable in order to compare to baseline. The parameters analyzed, the degrees of freedom (df), and the error degrees of freedom (df-error) are shown per experiment followed by their corresponding F-value and p-value.

Group	Data	Region	Parameter	df	df-error	F-value	p-value
0 h	ipsilateral	striatum	perfusion	3	16	23.73	< 0.001
			ADC	3	16	2.90	0.067
			T2	3	12	7.64	0.004
0 h	contralateral	striatum	perfusion	3	16	11.93	< 0.001
			ADC	3	16	2.55	0.092
			T2	3	12	4.321	0.028
0 h	ipsilateral	cortex	perfusion	3	16	29.66	< 0.001
			ADC	3	16	3.72	0.033
			T2	3	12	3.88	0.038
0 h	contralateral	cortex	perfusion	3	16	6.17	0.006
			ADC	3	16	1.01	0.416
			T2	3	12	0.88	0.479
BL / 96 h	ipsilateral	striatum	perfusion	7	31	8.194	< 0.001
			ADC	7	31	4.73	0.001
			T2	7	28	13.75	< 0.001
BL / 96 h	contralateral	striatum	perfusion	7	31	5.76	< 0.001
			ADC	7	31	1.45	0.222
			T2	7	28	2.45	0.043
BL / 96 h	ipsilateral	cortex	perfusion	7	31	4.71	0.001
			ADC	7	31	3.5	0.007
			T2	7	28	0.65	0.708
BL / 96 h	contralateral	cortex	perfusion	7	31	5.46	< 0.001
			ADC	7	31	1.51	0.201
			T2	7	28	4.02	0.004

Supplementary Table 3. 2-way analysis of variance (ANOVA) for MGE analysis. The table shows the degrees of freedom (df), the error degrees of freedom (df-error), and the corresponding F-value and p-value calculated for the 2- way ANOVA considering surgery type (Sham or Occlusion) and treatment (PTX or PBS).

**Data**	**Region**	**Parameter**	**df**	**df-error**	**F-value**	**p-value**
ipsilateral	cortex	perfusion	3	26	23.34	< 0.001
contralateral	cortex	perfusion	3	26	6.03	0.003
